# Neck and Waist Circumference as Correlates of Coronary Artery Disease: A Case-Control Analysis in a South Asian Population

**DOI:** 10.7759/cureus.99324

**Published:** 2025-12-15

**Authors:** Ashish Jindal, Vijay M Bengalorkar

**Affiliations:** 1 General Medicine, Dr. B.R. Ambedkar State Institute of Medical Sciences, Sahibzada Ajit Singh Nagar, IND; 2 General Medicine, Rajarajeswari Medical College and Hospital, Bengaluru, IND

**Keywords:** anthropometry, central obesity, coronary artery disease, neck circumference, risk factors, south asian, waist circumference

## Abstract

Background: The anatomical distribution of body fat is a critical determinant of cardiovascular risk. Simple anthropometric measures like neck circumference (NC) and waist circumference (WC) serve as proxies for upper-body and central adiposity.

Objectives: This study aimed to determine if NC and WC are independently associated with coronary artery disease (CAD) in a South Asian Indian cohort.

Methods: We conducted a case-control study at a tertiary care hospital in Bengaluru, India, including 100 patients with established CAD and 100 controls. Detailed anthropometric, clinical, and biochemical data were collected. Pearson's correlation and multivariate logistic regression analyses were used to assess the association of NC and WC with CAD and its related risk factors.

Results: Cases with CAD had significantly higher mean NC and WC than controls (p<0.001). In correlation analysis, both NC and WC were significantly and positively correlated with systolic and diastolic blood pressure, low-density lipoprotein cholesterol, and triglycerides, and negatively correlated with high-density lipoprotein cholesterol (p<0.001 for all). In a multivariate logistic regression model that included both anthropometric measures simultaneously to adjust for each other's effects, a 1-SD increase in NC (adjusted OR (aOR): 1.78; 95%CI: 1.32-2.30; p<0.001) and a 1-SD increase in WC (aOR: 2.15; 95%CI: 1.56-2.90; p<0.001) remained strong and mutually independent factors associated with CAD.

Conclusion: In this South Asian population, NC and WC are simple, inexpensive, and powerful correlates of CAD and its associated metabolic risk factors. Their mutual independence in statistical models suggests they represent distinct aspects of pathogenic adiposity, and their routine measurement can enhance cardiovascular risk assessment.

## Introduction

Coronary artery disease (CAD) remains a leading cause of morbidity and mortality globally, with a disproportionately high burden in South Asian populations, who often develop the disease at a younger age and with greater severity [1-3]. This elevated risk underscores the urgent need for effective and accessible risk stratification strategies tailored to this demographic.

The scientific consensus has shifted from viewing obesity as a monolithic condition measured by body mass index (BMI) to recognizing that the anatomical distribution of adipose tissue is a more crucial determinant of cardiometabolic risk. This is particularly relevant for South Asians, who often exhibit high body fat percentage at a "normal" BMI, a phenomenon termed the "Y-Y paradox" [4]. This "thin-fat" phenotype is well-documented in South Asian populations [5]. Consequently, reliance on BMI alone can fail to identify a substantial number of high-risk individuals.

Waist circumference (WC), a measure of central adiposity, is a well-established marker for CAD risk, a link confirmed as causal by large-scale studies like INTERHEART and subsequent Mendelian randomization analyses [6-8]. More recently, neck circumference (NC) has emerged as a reliable proxy for upper-body subcutaneous fat, a metabolically active depot that contributes to insulin resistance and dyslipidemia [9-11]. Data from the Framingham Heart Study demonstrated that NC is associated with an adverse cardiometabolic profile, even after adjusting for BMI and visceral fat, suggesting it captures a unique aspect of risk [9,12].

This creates a critical need to validate simple clinical tools like NC and WC. While the individual associations of NC and WC with CAD are documented, their concurrent independent value within a single, fully adjusted model has not been robustly established in a high-risk South Asian cohort. Building on evidence distinguishing upper-body from visceral fat depots [12,13], this study was designed to address this gap by simultaneously testing NC and WC in a single model to determine if each provides unique, non-redundant information regarding CAD risk. This is important because NC and WC capture different fat depots (upper body vs. abdominal), and finding both independently associated with CAD would suggest that combined measurement could enhance risk stratification [14]. Furthermore, South Asian individuals often exhibit increased visceral adiposity even at relatively low BMI, underscoring the need to study both central (WC) and upper-body (NC) adiposity in this population [5].

## Materials and methods

This hospital-based case-control study was conducted at the Rajarajeswari Medical College and Hospital, a tertiary care center in Bengaluru, India, between January 2016 and December 2018. The study protocol adhered to the STROBE (Strengthening the Reporting of Observational Studies in Epidemiology) guidelines [15]. The study was approved by the Institutional Ethics Committee of Rajarajeswari Medical College and Hospital and conducted in accordance with the Declaration of Helsinki [16]. All participants provided written informed consent.

Study population

Eligibility Criteria

Cases were patients with established CAD, confirmed by clinical evaluation, electrocardiography (ECG), and/or coronary angiography. Controls were recruited from the local community (e.g., hospital visitors without cardiovascular symptoms) and had no history or symptoms of CAD, confirmed by a normal resting ECG.

Exclusion criteria were a known diagnosis of Type 1 or Type 2 diabetes mellitus, adherence to a strict diet regimen, use of weight reduction medications, and refusal to provide written informed consent. 

Sample Size

**Figure 1:**
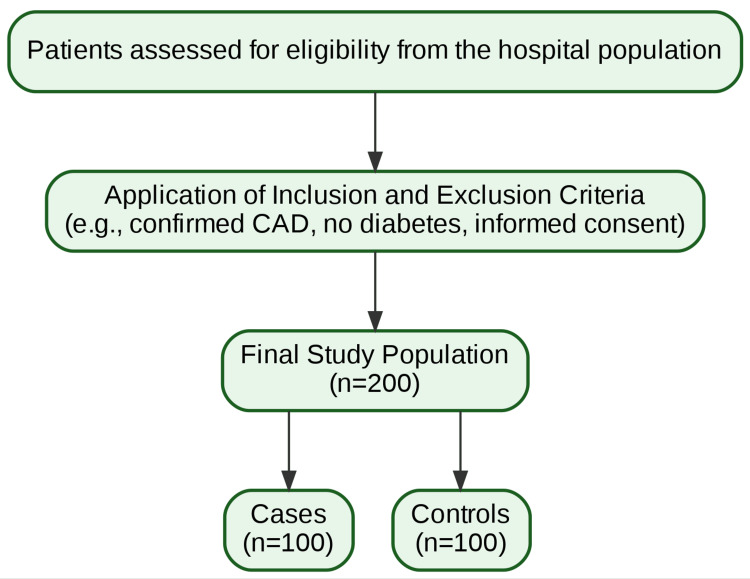
Study flow diagram. The flowchart illustrates the process of participant selection for the case-control study, showing the final enrollment of 100 CAD cases and 100 controls. CAD: coronary artery disease

The study included 100 patients with a confirmed diagnosis of CAD (cases) and 100 individuals without clinical evidence of CAD (controls). A formal a priori sample size calculation was not performed; however, we enrolled 100 cases and 100 controls based on study feasibility, and subsequent post-hoc analysis confirmed that the sample was more than adequately powered to detect the observed effect sizes. The groups were matched by age and sex. A statistically significant difference in mean age was observed between female cases and controls, which was accounted for by including age as a covariate in all multivariate models. The participant selection process is summarized in Figure 1.

Data collection and clinical measurements

NC was measured with the participant standing, head erect, at the level of the superior border of the thyroid cartilage, a method consistent with large-scale cardiometabolic studies [6]. WC was measured at the midpoint between the lower margin of the last palpable rib and the top of the iliac crest, aligning with the standardized method used in the INTERHEART study [6]. Each anthropometric measurement (NC and WC) was taken twice, and the mean of the two values was used for analysis to improve measurement reliability.

Blood Pressure was measured in the right arm with the participant seated after a five-minute rest; two readings were taken, and the average of these readings was used for analysis. All anthropometric measurements were performed by trained personnel blinded to participants’ case/control status to minimize measurement bias. Inter- and intra-observer variability for NC and WC measurements was not formally assessed.

Data on smoking status, physical activity, and medication use were not collected in this study, which are potential confounders for CAD and adiposity [6].

Biochemical analysis

Venous blood samples were collected after an overnight fast. Serum lipid profiles were analyzed using a semi-automated clinical chemistry analyzer (Erba Chem 7, Transasia Bio-Medicals Ltd., Mumbai, Maharashtra, India) with standardized enzymatic colorimetric kits (Erba Mannheim, Dubai, United Arab Emirates). Biochemical assays were performed using standardized protocols with routine internal quality controls as per manufacturer guidelines.

Statistical analysis

Data were analyzed using R software, version 4.3.2 (R Foundation for Statistical Computing, Vienna, Austria, https://www.R-project.org/). Continuous variables were assessed for normality (e.g., Shapiro-Wilk test) and compared using Student’s t-test (or non-parametric tests if normality was violated). Pearson's correlation coefficient (r) was calculated to assess the linear relationship between anthropometric measures and continuous cardiometabolic variables. To assess the independent association with CAD, binary logistic regression was performed, with odds ratios (ORs) calculated per 1 SD increase. The final multivariable model was adjusted for age, sex, systolic blood pressure (SBP), diastolic blood pressure (DBP), low-density lipoprotein (LDL) cholesterol, high-density lipoprotein (HDL) cholesterol, and triglycerides (TG), and importantly, included both NC and WC simultaneously to test for mutual independence. Variance inflation factors (VIFs) were calculated to assess multicollinearity between NC and WC in the regression model. A p-value of <0.05 was considered statistically significant. No missing data were observed for key variables; analyses were conducted on complete cases only. No adjustments were made for multiple comparisons, given the exploratory nature of the analysis.

## Results

Baseline characteristics

**Figure 2:**
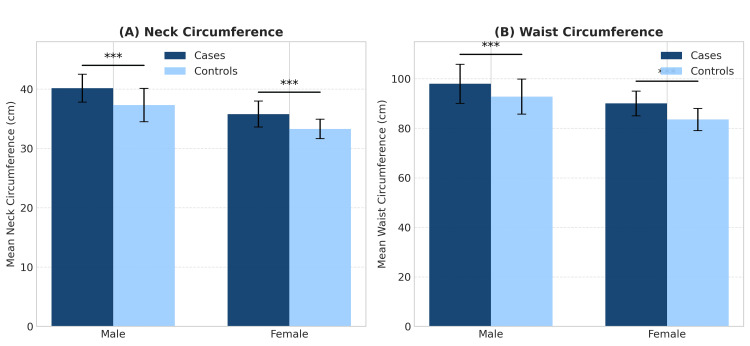
Comparison of mean anthropometric measurements between male and female sexes in cases and controls. Error bars represent the standard deviation. Asterisks (***) indicate a statistically significant difference (p<0.001) between cases and controls within each sex.

The baseline characteristics of the 200 participants are summarized in Table 1. All continuous variables were approximately normally distributed (Shapiro-Wilk p>0.05), justifying the use of parametric tests. The CAD group had significantly higher mean values for NC, WC, SBP, DBP, LDL, and TG, and significantly lower mean HDL levels compared to the control group (p<0.001 for all) (Figure 2). 

**Table 1:** Baseline characteristics of study participants Data are presented as n (%) or mean ± SD. Statistical test used: Independent Student's t-test. The t-value column reports the t-statistic, and the p-value corresponds to this statistic. NS: not significant; BP: blood pressure; LDL: low-density lipoprotein; HDL: high-density lipoprotein

Characteristics	Cases (n=100)	Controls (n=100)	t-value	p-value
Demographics				
Male Sex, n (%)	65 (65.0)	65 (65.0)	—	NS
Female Sex, n (%)	35 (35.0)	35 (35.0)	—	NS
Age (years), mean ± SD				
Male	57.80 ± 12.61	58.08 ± 6.98	-0.151	NS
Female	63.54 ± 11.54	53.71 ± 7.72	4.398	<0.01
Anthropometrics				
Neck Circumference (cm), mean ± SD				
Male	40.14 ± 2.35	37.30 ± 2.81	6.262	<0.001
Female	35.79 ± 2.18	33.29 ± 1.63	5.922	<0.001
Waist Circumference (cm), mean ± SD				
Male	97.98 ± 7.89	92.80 ± 7.11	3.864	<0.001
Female	90.03 ± 5.01	83.55 ± 4.45	6.076	<0.001
Clinical & Biochemical				
Systolic BP (mmHg), mean ± SD	133.25 ± 13.85	125.45 ± 12.10	3.999	<0.001
Diastolic BP (mmHg), mean ± SD	83.49 ± 10.58	78.80 ± 9.95	3.098	<0.001
LDL (mg/dL), mean ± SD	152.59 ± 29.98	102.15 ± 25.50	12.011	<0.001
HDL (mg/dL), mean ± SD	39.67 ± 7.11	44.50 ± 5.25	-4.958	<0.001
Triglycerides (mg/dL), mean ± SD	109.34 ± 30.75	95.60 ± 22.40	3.197	<0.01

Correlation analyses

**Figure 3:**
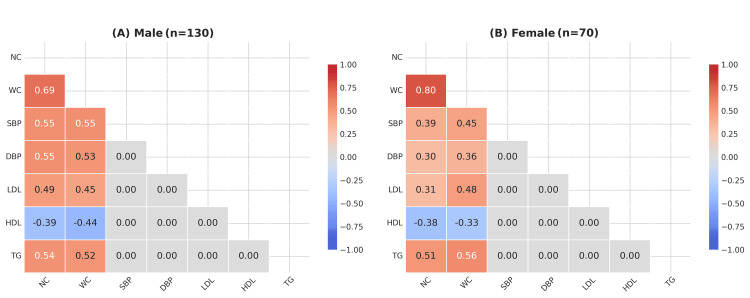
Heatmaps displaying the Pearson correlation of anthropometric measures with cardiometabolic risk factors startified by sex. NC: neck circumference; WC: waist circumference; LDL: low-density lipoprotein; HDL: high-density lipoprotein; SBP: systolic blood pressure; DBP: diastolic blood pressure; TG: triglycerides

As shown in Table 2 and Figure 3, Pearson's correlation analysis revealed significant associations between anthropometric measures and other cardiometabolic risk factors. In both male and female participants, NC and WC demonstrated a strong positive correlation with each other. Furthermore, both NC and WC were positively and significantly correlated with SBP, DBP, LDL, and TG, and were negatively correlated with HDL (p<0.001 for most variables). No formal adjustment for multiple testing was applied, so the significant correlations should be interpreted cautiously.

**Table 2:** Pearson's correlation of anthropometric measures with cardiometabolic risk factors. Statistical test used: Pearson's correlation. Data represents the test statistic (correlation coefficient r) and the corresponding p-value. Data for analysis derived from the original study's master chart, combining cases and controls for each sex. LDL: low-density lipoprotein; HDL: high-density lipoprotein; BP: blood pressure

Parameters	Neck Circumference (cm)	Waist Circumference (cm)
Male (n=130)		
Waist Circumference (cm)	r = 0.686 (p<0.001)	-
Systolic BP (mmHg)	r = 0.554 (p<0.001)	r = 0.550 (p<0.001)
Diastolic BP (mmHg)	r = 0.547 (p<0.001)	r = 0.530 (p<0.001)
LDL (mg/dL)	r = 0.489 (p<0.001)	r = 0.446 (p<0.001)
HDL (mg/dL)	r = -0.390 (p<0.001)	r = -0.441 (p<0.001)
Triglycerides (mg/dL)	r = 0.539 (p<0.001)	r = 0.522 (p<0.001)
Female (n=70)		
Waist Circumference (cm)	r = 0.795 (p<0.001)	-
Systolic BP (mmHg)	r = 0.392 (p<0.001)	r = 0.449 (p<0.001)
Diastolic BP (mmHg)	r = 0.303 (p=0.001)	r = 0.360 (p<0.001)
LDL (mg/dL)	r = 0.307 (p=0.001)	r = 0.484 (p<0.001)
HDL (mg/dL)	r = -0.376 (p<0.001)	r = -0.329 (p=0.001)
Triglycerides (mg/dL)	r = 0.511 (p<0.001)	r = 0.564 (p<0.001)

Association of anthropometric measures with coronary artery disease

The results of the logistic regression analysis are presented in Table 3. In the final, fully adjusted model that included both NC and WC to account for each other's effects, both measures remained strong and statistically significant factors associated with CAD. No significant interactions by sex were observed (p>0.1), and correlation patterns were similar for males and females. Correlation analyses in cases versus controls yielded similar patterns (data not shown), suggesting that disease status did not materially alter these associations. The VIF for NC and WC was 1.89 in male and 2.70 in female participants, both well below the commonly accepted threshold of 5, indicating acceptable multicollinearity and supporting inclusion of both variables in the model. A 1-SD increase in NC was associated with a 78% increase in the odds of having CAD (adjusted OR (aOR): 1.78; 95%CI: 1.32-2.30; p<0.001), while a 1 SD increase in WC was associated with more than double the odds of having CAD (aOR: 2.15; 95%CI: 1.56-2.90; p<0.001). Here, 1 SD refers to the standard deviation of NC or WC calculated from the combined sample of cases and controls.

**Table 3:** Multivariate logistic regression analysis of anthropometric measures as factors associated with coronary artery disease Statistical test used: Multivariate Binary Logistic Regression. p-values correspond to the Wald z-statistic. *Adjusted OR is adjusted for age, sex, systolic blood pressure, diastolic blood pressure, low-density lipoprotein cholesterol, high-density lipoprotein cholesterol, triglycerides, and includes both neck circumference and waist circumference in the same model.

Associated Factor (per 1 SD increase)	Crude OR (95% CI)	p-value	Adjusted OR* (95% CI)	p-value
Neck Circumference	2.05 (1.52–2.76)	<0.001	1.78 (1.32–2.30)	<0.001
Waist Circumference	2.41 (1.75–3.32)	<0.001	2.15 (1.56–2.90)	<0.001

## Discussion

In this case-control study of a South Asian Indian population, we found that NC and WC are strong and independent correlates of coronary artery disease. The study's primary contribution to the literature is the demonstration that NC and WC are mutually independent associated factors. When both measures were included simultaneously in a fully adjusted regression model, each retained its strong, significant association with CAD. This novel finding suggests that these two simple measures are not redundant proxies for general obesity but rather represent distinct regional fat depots (upper-body subcutaneous fat for NC and visceral abdominal fat for WC) that are each independently associated with the atherogenic risk profile in this high-risk population.

Our findings on WC reinforce the critical role of central adiposity in atherogenesis, aligning with landmark studies like INTERHEART and causal evidence from Mendelian randomization studies [6-8]. The strong, independent association of NC is equally important, supporting research from the Framingham Heart Study that identified upper-body fat as a unique, pathogenic depot [9,11]. The new correlation analyses (Table 2) further strengthen the study by showing that both NC and WC are not just associated with the presence of CAD, but are also significantly correlated with the individual metabolic disturbances that drive the disease. These observed associations parallel the metabolic risk clustering reported in recent global and regional cohorts [1,17,18].

The clinical utility of these findings is significant. NC and WC are simple, non-invasive, and inexpensive tools that can be easily integrated into routine screening. Their demonstrated independent association with CAD suggests that measuring both could provide a more comprehensive risk assessment than using either one alone, helping to identify at-risk individuals who may benefit from more intensive preventive strategies.

Limitations

This study has several limitations that must be acknowledged. First, the case-control design establishes association but cannot infer causality or temporal relationships; therefore, we have referred to NC and WC as "associated factors" or "correlates" rather than "predictors." A prospective study would be required to establish predictive value. Second, as a single-center study, the findings may have limited generalizability. Third, the deliberate exclusion of patients with known diabetes, while strengthening internal validity by removing a powerful confounder, limits the external validity of our findings to the large and important population of patients with both CAD and diabetes. Fourth, we did not collect data on other important risk factors such as smoking status or family history of cardiovascular disease. This is a significant limitation because smoking is a major CAD risk factor that could confound the observed associations between anthropometric measures and CAD [6]. Additionally, selecting controls based on a normal ECG may have failed to exclude subclinical CAD, leading to possible misclassification bias. Because our cases already had established CAD, we cannot rule out reverse causation (e.g., if CAD or its treatment affected body composition), which should be acknowledged. The significant age imbalance among female participants was adjusted for in the regression models; however, residual confounding by age or other factors cannot be excluded without stratified sensitivity analyses. Finally, our study did not include newer anthropometric indices, such as the waist-to-height ratio, which some studies suggest may be a superior marker of cardiometabolic risk in South Asian populations and should be a focus of future research [5,19,20]. Future studies should establish population-specific cut-off values for NC and WC and assess whether adding both measures to risk models improves CAD prediction, using prospective cohorts [17].

## Conclusions

This study provides strong evidence that in a South Asian population, NC and WC are powerful, independent correlates of coronary artery disease. The mutual independence of these simple measures suggests they capture unique aspects of pathogenic adiposity. If confirmed in prospective studies, combined measurement of NC and WC could potentially enhance early identification of individuals at high cardiovascular risk. Future research is needed to determine optimal cut-off values for NC and WC in this population and to evaluate their combined predictive value for CAD in diverse South Asian cohorts.
